# Predicting Conversion From Laparoscopic to Open Cholecystectomy: A Prospective Risk Factor Analysis and Scoring Model Formulation

**DOI:** 10.7759/cureus.91916

**Published:** 2025-09-09

**Authors:** Shesh Kumar, Ramlakhan S Verma, Rafey A Rahman, Somendra Pal Singh, Atif Khan

**Affiliations:** 1 General Surgery, Uttar Pradesh University of Medical Sciences, Etawah, IND; 2 Pediatric Surgery, Uttar Pradesh University of Medical Sciences, Etawah, IND

**Keywords:** adhesions, conversion, laparoscopic cholecystectomy, open cholecystectomy, risk factors, scoring system

## Abstract

Background

Laparoscopic cholecystectomy (LC) is the gold standard treatment for symptomatic gallstone disease. However, a subset of cases requires conversion to open cholecystectomy (OC) due to anatomical challenges or intraoperative complications.

Objective

To identify the preoperative and intraoperative risk factors associated with conversion of LC to OC and propose a simple scoring system to predict high-risk cases.

Methodology

A prospective observational study was conducted in the Department of General Surgery, Uttar Pradesh University of Medical Sciences, Saifai, Etawah, a tertiary center in Northern India serving mainly a rural population, from June 2023 to December 2024. Patients undergoing LC for symptomatic cholelithiasis were enrolled. Preoperative variables, including age, gender, comorbidities, previous abdominal surgery, and laboratory markers, were recorded. Intraoperative findings, such as gallbladder appearance, adhesions, Calot’s triangle anatomy, presence of common bile duct (CBD) stones, cholecystoenteric fistula, and attainment of the critical view of safety (CVS), were analyzed. Statistical significance was set at *P *< 0.05.

Results

Of 318 patients, 33 (10.38%) required conversion to OC. Significant preoperative risk factors included previous abdominal surgery (*P *= 0.047) and elevated alkaline phosphatase (ALP) levels (*P *< 0.001). Intraoperative predictors such as a contracted gallbladder (*P *< 0.001), dense pericholecystic adhesions (*P *< 0.001), non-visualization of Calot’s triangle (*P *< 0.001), failure to attain CVS (*P *< 0.001), CBD stones (*P *< 0.001), and cholecystoenteric fistula (*P *< 0.001) were strongly associated with conversion. A weighted scoring system was developed for risk stratification.

Conclusions

Conversion from laparoscopic to OC, though infrequent (10.38%), is crucial for patient safety in selected cases. Prior abdominal surgery and elevated alkaline phosphatase predicted conversion preoperatively, while dense adhesions, poor Calot’s triangle visualization, failure to achieve the critical view of safety, cholecystoenteric fistula, and CBD stones were key intraoperative determinants. The proposed risk scoring system enables early identification of high-risk patients, improving surgical planning, decision-making, and outcomes.

## Introduction

Gallstone disease is one of the most common gastrointestinal disorders requiring surgical intervention, often presenting with abdominal pain, dyspepsia, and related biliary symptoms [1]. Cholecystectomy remains the definitive treatment for symptomatic cholelithiasis, with laparoscopic cholecystectomy (LC) now the gold standard owing to its advantages over open cholecystectomy (OC), including reduced postoperative pain, shorter hospital stay, faster recovery, and lower morbidity and mortality rates [2-4].

Despite these benefits, conversion from LC to OC remains an essential intraoperative safety measure in selected cases. Reported conversion rates range from 1% to 24%, influenced by patient characteristics, disease severity, surgeon experience, and intraoperative findings [3,4]. Conversion is associated with longer operative time, increased blood loss, higher rates of surgical site infection, prolonged hospitalization, and greater healthcare costs. Delayed or inappropriate conversion may also increase the risk of major complications, including bile duct injury [5,6].

Multiple preoperative and intraoperative factors have been implicated in predicting conversion. Preoperative predictors include advanced age, male sex, obesity, diabetes, prior upper abdominal surgery, and laboratory or imaging findings such as elevated alkaline phosphatase (ALP), gallbladder wall thickening, and pericholecystic fluid [7-9]. Intraoperative predictors include dense adhesions, unclear Calot’s triangle anatomy, inability to achieve the critical view of safety (CVS), common bile duct (CBD) stones, and cholecystoenteric fistula [10]. Early identification of these factors allows better surgical planning, informed patient consent, and optimized allocation of operative resources.

Existing literature on predictors of conversion is largely retrospective, often limited by incomplete data and heterogeneous definitions [11,12]. For example, studies vary in their definition of *conversion* (conversion at any stage vs. only after failed dissection), criteria for intraoperative difficulty (subjective grading vs. standardized scales), and laboratory thresholds (e.g., ALP >120 IU/L vs. >150 IU/L). While several systematic reviews and meta-analyses, including the recent ones, have attempted to synthesize the evidence, variations in surgical techniques, perioperative protocols, and imaging modalities limit generalizability [13-15]. Furthermore, few prospective studies, particularly in resource-limited settings, have developed and validated practical scoring models for predicting conversion.

The present study was undertaken to assess both preoperative and intraoperative risk factors associated with conversion from LC to OC and to propose a predictive risk scoring system for use in clinical practice.

## Materials and methods

Study design and setting

This was a single-center, prospective observational cohort study conducted in the Department of General Surgery, Uttar Pradesh University of Medical Sciences, Saifai, Etawah, a tertiary center in Northern India serving mainly a rural population, from June 2023 to December 2024, after approval from the Institutional Ethics Committee (IEC No. 50/2023-24).

Patient selection

All consecutive patients undergoing elective LC for symptomatic cholelithiasis were enrolled after written informed consent. Patients with acute cholecystitis requiring emergency surgery, gallbladder malignancy, or lack of consent were excluded. Emergency cases were excluded to avoid heterogeneity, as acute presentations involve different inflammatory severity, urgency of decision-making, and risk profiles compared with elective surgery.

Conversion was defined as any intraoperative switch from laparoscopic to open technique, including subtotal cholecystectomy performed when safe dissection of Calot’s triangle was not feasible.

Data collection

After providing informed consent, patients were evaluated through history, physical examination, and laboratory investigations, including complete blood count (CBC), liver function test (LFT), random blood sugar (RBS), renal function test (RFT), serum electrolytes, and viral markers. Preoperative factors assessed included age, gender, comorbidities (hypertension, diabetes, chronic obstructive pulmonary disease, and cardiovascular disease), previous abdominal surgery, serum ALP, and gallbladder wall thickness. Intraoperative findings assessed included gallbladder appearance (distended vs. contracted), pericholecystic adhesions (minimal vs. dense), Calot’s triangle anatomy (defined vs. not defined), attainment of CVS, presence of CBD stones, and presence of cholecystoenteric fistula.

All procedures were performed by a consistent surgical team using the standard four-port technique for LC. Conversion to OC was decided by the operating surgeon based on safety and feasibility.

Statistical analysis

Data were analyzed using statistical software SPSS (version 30.0, IBM Corp., Armonk, NY). Continuous variables (e.g., age, ALP levels) were analyzed using independent t-tests, while categorical variables were compared using chi-square tests. A *P*-value <0.05 was considered statistically significant. Odds ratios with 95% confidence intervals were calculated for significant predictors.

For the scoring model, weight assignment was based on clinical judgment supported by univariate significance levels rather than regression coefficients. Each variable was assigned a weighted score according to its relative importance and statistical association. This preliminary scoring system is exploratory and requires external validation through regression analysis and receiver operating characteristic (ROC) curve testing in future studies.

## Results

A total of 318 patients underwent LC, of which 33 cases required conversion to OC, resulting in an overall conversion rate of 10.38% (Table 1).

**Table 1:** Distribution of cases according to conversion status.

Conversion status	n	%
Laparoscopic cholecystectomy	285	89.62
Converted to an open procedure	33	10.38

Preoperative factors

We observed that younger patients had relatively higher conversion rates, with a statistically significant association with age (*P* = 0.043). In contrast, gender did not show a significant association (*P* = 0.123). Common comorbidities, including hypertension, diabetes mellitus, chronic obstructive pulmonary disease (COPD), and cardiovascular disease, were also not significantly correlated with conversion. Among preoperative variables, a history of previous abdominal surgery showed a significant association (*P* = 0.047), indicating that patients with prior surgical interventions were more likely to require conversion. Notably, elevated preoperative serum ALP levels were strongly associated with conversion (*P *< 0.001), suggesting their potential role as a predictive preoperative biomarker. Table 2 summarizes the associations between various preoperative variables and conversion of LC to OC.

**Table 2:** Associations between various preoperative variables and conversion of laparoscopic cholecystectomy to open procedure. COPD, chronic obstructive pulmonary disease; CVD, cardiovascular disease; ALP, alkaline phosphatase; SD, standard deviation

Variable	Category	Laparoscopic cholecystectomy (*n *= 285)	Converted to open procedure (*n *= 33)	Statistical test	*P*-value
Age (years)					
	Mean ± SD	38.67 ± 13.10	34.73 ± 9.94	*t*-test (*t *= 2.08)	0.043
Gender, *n* (%)					
	Male	43 (15.09%)	9 (27.27%)	Chi-square (*χ*² = 2.38)	0.123
	Female	242 (84.91%)	24 (72.73%)		
Comorbidities, *n* (%)					
	Hypertension (HTN)	51 (17.89%)	7 (21.21%)	Chi-square (χ² = 0.22)	0.64
	Type 2 diabetes mellitus (T2DM)	17 (5.96%)	3 (9.09%)	Chi-square (χ² = 0.49)	0.484
	COPD	2 (0.70%)	1 (3.03%)	Chi-square (χ² = 1.72)	0.19
	Other cardiovascular diseases	7 (2.46%)	0 (0.00%)	Chi-square (χ² = 0.83)	0.363
Previous abdominal surgery, *n* (%)					
	Yes	19 (6.7%)	6 (18.2%)	Chi-square (χ² = 3.94)	0.047
	No	266 (93.3%)	27 (81.8%)		
Preoperative ALP (IU/L)					
	Mean ± SD	124.40 ± 18.79	159.21 ± 60.59	t-test (*t *= -7.212)	<0.001

Intraoperative factors

Regarding intraoperative findings, a contracted gallbladder was observed significantly more frequently in the converted group (13, 39.39%) compared to the laparoscopic group (35, 12.28%) (*P *< 0.001). Dense pericholecystic adhesions were noted in 29 (87.88%) converted cases compared with 25 (8.77%) cases completed laparoscopically, making this one of the most significant predictors of conversion (*P* < 0.001). Cholecystoenteric fistulae were present exclusively in the converted group (6, 18.18%), with no occurrences in the laparoscopic group, representing a highly significant association (*P* < 0.001).

Anatomical challenges also played a major role. Calot’s triangle was not clearly defined in 23 (69.70%) converted cases compared with 61 (21.40%) cases in the laparoscopic group (*P* < 0.001). Additionally, failure to achieve the CVS was significantly more frequent in the converted group (81.82% vs. 40.70%, *P* < 0.001). The presence of CBD stones was another important predictor, found in 4 (12.12%) converted cases versus 1 (0.35%) in the laparoscopic group (*P* < 0.001). Association of intraoperative variables with conversion to OC is depicted in Table 3.

**Table 3:** Association of intraoperative variables with conversion to open cholecystectomy (n = 318). The chi-square test was used to calculate the *P*-value. CBD, common bile duct

Intraoperative parameter	Variable	Laparoscopic (*n* = 285)	Converted (*n* = 33)	Chi-square	*P*-value	Odds ratio
Gallbladder appearance	Distended	250 (87.72%)	20 (60.61%)	16.9	<0.001	8.68
	Contracted	35 (12.28%)	13 (39.39%)			
Pericholecystic adhesions	Minimal	260 (91.23%)	4 (12.12%)	125.73	<0.001	88.09
	Dense	25 (8.77%)	29 (87.88%)			
Cholecystoenteric fistula	Present	0 (0.00%)	6 (18.18%)	52.81	<0.001	-
	Absent	285 (100.00%)	27 (81.82%)			
Calot’s triangle anatomy	Defined	224 (78.60%)	10 (30.30%)	35.49	<0.001	8.57
	Not defined	61 (21.40%)	23 (69.70%)			
Critical view of safety (CVS)	Achieved	169 (59.30%)	6 (18.18%)	19.1	<0.001	12.05
	Not achieved	116 (40.70%)	27 (81.82%)			
CBD stones	Present	1 (0.35%)	4 (12.12%)	26.48	<0.001	-
	Absent	284 (99.65%)	29 (87.88%)			

Extent of cholecystectomy

Finally, only 36.36% of the converted cases underwent total cholecystectomy, in contrast to 100% of cases completed laparoscopically, indicating that subtotal cholecystectomy was more frequently performed in technically challenging conversions.

## Discussion

The present study observed a conversion rate of 10.38%, which is consistent with previously reported rates in the literature, ranging from 2% to 15% [14,16]. This finding underscores the continuing relevance of conversion to OC in the laparoscopic era. While laparoscopy remains the gold standard for cholecystectomy, certain clinical and anatomical challenges necessitate conversion to ensure patient safety and operative success.

Conversion rates and predictive factors

The observed conversion rate of 10.38% aligns with various studies reported in literature [14,16,17,18]. Among preoperative factors, a history of previous abdominal surgery was significantly associated with conversion (*P *= 0.047). This is supported by findings from the study of Wakabayashi et al. [13] and Pavlidis et al. [17], which also highlight adhesions from prior surgeries as a frequent impediment to safe dissection.

Elevated preoperative ALP levels were another significant predictor (*P *< 0.001). This result is consistent with studies by Lipman et al. [19] and Liu et al. [20], who emphasized biochemical markers as potential surrogates for inflammation, biliary obstruction, or difficult anatomy.

Although both younger age and male gender showed a trend toward higher conversion rates, only the association with age reached statistical significance in our study, while gender did not. In contrast, previous studies, including those by Magnano San Lio et al. [15] and Akcakaya et al. [21], reported higher conversion rates in males, possibly attributable to delayed presentation and more advanced disease.

Comorbidities, including hypertension, type 2 diabetes, COPD, and cardiovascular diseases, did not significantly affect conversion rates, in line with the observations of Rothman et al. [14]. Contrarily, Coaston et al. [22] and Kadirvel et al. [23] found diabetes and obesity to be significant predictors, indicating regional or methodological variation in risk profiling.

Intraoperative risk factors

Intraoperative findings had a more profound association with conversion. A contracted gallbladder, dense pericholecystic adhesions, and cholecystoenteric fistulae were all significantly more common in converted cases. These findings mirror the experiences reported in various other studies [24-26].

A particularly striking finding was the association of anatomical challenges with conversion. Undefined Calot’s triangle anatomy and failure to achieve the CVS were more frequent among converted cases (81.8% vs. 40.7%, *P* < 0.001). These are widely acknowledged markers of technical difficulty and risk for iatrogenic injury. Studies by Rothman et al. [14] and Alius et al. [27] emphasize that attaining the CVS is central to safe cholecystectomy.

Additionally, the presence of CBD stones significantly increased the likelihood of conversion (*P *< 0.001), reinforcing their importance in preoperative evaluation and intraoperative decision-making, as described by Lipman et al. [19] and Liu et al. [20].

An important observation was that subtotal cholecystectomy was more commonly performed in converted cases, reflecting its use as a bailout strategy in technically challenging scenarios. This aligns with previous reports highlighting subtotal cholecystectomy as a safe alternative to total cholecystectomy in preventing bile duct injury when dissection is hazardous [28,29]. Outcomes of bailout approaches, including reduced rates of major biliary complications, support its role in difficult cases.

Scoring system and clinical implications

To facilitate early risk identification and guide intraoperative decision-making, we proposed a weighted risk scoring system incorporating both preoperative and intraoperative variables (Table 4). High-scoring patients (≥10) may benefit from advanced planning, early senior involvement, or a lower threshold for conversion. This aligns with models such as the G10 score and Nassar’s difficulty grading scale [30]. The practical utility of such a scoring system includes facilitating preoperative patient counselling, intraoperative team readiness, avoiding delays in necessary conversion, and potentially reducing complication rates. It should be noted that this scoring system was internally developed within our cohort and has not been externally validated.

**Table 4:** Risk scoring system incorporating both preoperative and intraoperative variables. Scores (≥10): Advanced planning, early senior involvement, and a lower threshold for conversion.

Factor	Criteria	Score
Previous abdominal surgery	No	0
	Yes	2
Calot’s triangle anatomy	Well defined	0
	Not defined	3
Critical view of safety attained	Yes	0
	No	3
Pericholecystic adhesions	None/mild	0
	Dense	3
Cholecystoenteric fistula	Absent	0
	Present	4
Preoperative alkaline phosphatase (ALP) levels	<150 IU/L	0
	>150 IU/L	2
Intraoperative gallbladder appearance	Distended	0
	Contracted	2
Common bile duct (CBD) stones	Absent	0
	Present	3
Age	<60 years	0
	≥60 years	1
Gender	Female	0
	Male	1

Limitations

This study has several limitations. It was conducted at a single center by a single surgical team, which may limit generalizability but ensures procedural consistency. The absence of a formal sample size calculation is another limitation. Laboratory variability, such as differences in ALP cutoffs across institutions, may affect external applicability. Furthermore, our scoring system was formulated based on univariate analysis and clinical judgment and has not undergone external validation. Future multicenter studies with multivariate analysis and ROC validation are required to refine and validate the scoring system.

## Conclusions

Conversion from LC to OC remains an essential intraoperative decision to safeguard patient safety and achieve optimal outcomes. In this prospective observational study, the conversion rate was 10.38%, with significant predictors identified in both preoperative and intraoperative domains. Prior abdominal surgery and elevated serum ALP were key preoperative predictors, whereas dense pericholecystic adhesions, non-visualization of Calot’s triangle, failure to achieve the CVS, cholecystoenteric fistula, and CBD stones emerged as the most influential intraoperative determinants.

These findings emphasize the importance of early recognition of high-risk features and the need for proactive surgical strategies. The proposed risk scoring system provides a practical framework for early risk identification and surgical planning. However, the scoring system is preliminary and requires external validation before widespread adoption in clinical practice.
